# Ultrasound-Guided Suprainguinal Fascia Iliaca Block as Part of Anesthesia Management for Lower Extremity Surgeries: A Single-Center Retrospective Cohort Feasibility Study

**DOI:** 10.7759/cureus.47795

**Published:** 2023-10-27

**Authors:** Caner Genc, Sevda Akdeniz, Senay Canikli, Hatice Selcuk Kusderci, Hale Kefeli Celik, Serkan Tulgar

**Affiliations:** 1 Department of Anesthesiology and Reanimation, Samsun University Faculty of Medicine, Samsun Training and Research Hospital, Samsun, TUR

**Keywords:** surgical anesthesia, anesthesia management, ultrasound-guided, orthopedic surgery, anesthesia, nerve block, supra-inguinal fascia iliaca block

## Abstract

Study objective

The ultrasound-guided (US-guided) suprainguinal fascia iliaca block (SIFIB) is a regional anesthesia procedure that targets the lumbar plexus. It offers versatility in clinical practice, serving as both a standalone method for adequate pain management and a primary anesthesia option. Our aim was to present clinical insights gained from the application of US-guided SIFIB, whether as a standalone procedure or in conjunction with another block, across various clinical indications for lower extremity surgeries.

Methodology

Our study is a retrospective cohort analysis designed to identify cases in which the SIFIB was used as a component of the main anesthetic method and to determine the success of the anesthetic method in patients undergoing lower extremity surgery between March 2022 and March 2023 in a tertiary hospital. Data such as block success, perioperative additional analgesic need, patients' demographic details, and block characteristics were obtained from electronic and paper-based patient records and analyzed.

Main results

We analyzed data from 16 patients who underwent lower extremity surgeries under SIFIB. Among these, 10 patients received SIFIB as their sole anesthesia method, while six underwent surgery with a combination of sciatic block and SIFIB. Briefly, the types of surgery were amputations, soft tissue excision, revision of knee prostheses, excision of knee tumors, patella implant removal, patellar ligament repair, patellar fracture repair, distal femur fractures (internal fixation), and vascular surgery. Six patients necessitated additional analgesics. No statistically significant differences were observed in demographic details, block onset time, and surgical duration between patients requiring and not requiring sedoanalgesia during surgery (p>0.05).

Conclusion

For patients planning lower extremity surgery, considering SIFIB alone or combined with a sciatic block as part of anesthesia management is a valid option, offering an alternative to a lumbar plexus block.

## Introduction

Orthopedic procedures have become increasingly prevalent in clinical practice. Notably, the landscape of perioperative pain management and anesthesia strategies has undergone significant advancement in recent years. The adoption of regional anesthesia techniques, often coupled with multimodal analgesic approaches, has ushered in a transformative era, substantially diminishing the reliance on general anesthesia across a wide spectrum of surgical interventions. Pain management strategies for lower extremity surgeries encompass a range of techniques, neuraxial anesthesia, and occasionally, peripheral nerve blocks and interfascial plane blocks or combinations thereof, emerging as effective and commonly employed approaches [1]. Within the realm of nerve blocks, a spectrum of options includes selective nerve and plexus blocks, such as those targeting the lumbar plexus, femoral nerve, obturator nerve, and sciatic nerve at different anatomical levels. Additionally, fascial plane blocks like the fascia iliaca block represent an array of choices available. Importantly, these techniques serve a dual purpose, finding application not only in providing effective analgesia but also in serving as anesthesia methods, thereby offering a versatile approach to pain management in clinical practice.

The fascia iliaca block was initially described by Dalens et al. [2] in 1989, with the suprainguinal approach to the fascia iliaca being expounded upon by Stevens et al. [3] in 2007. The definition of the ultrasound-guided (US-guided) suprainguinal fascia iliaca block (SIFIB) was established by Hebbart et al. [4]. Randomized controlled trials have performed comparative analyses between SIFIB and the traditional fascia iliaca block technique, consistently demonstrating that SIFIB is associated with improved postoperative outcomes [5,6]. Consequently, it finds utility in clinical practice as part of multimodal analgesia strategies.

Our aim in this proof-of-concept retrospective study was to investigate the feasibility of applying SIFIB as the main anesthetic method, alone or in combination with a sciatic block, in a range of clinical indications for lower extremity surgeries and to present the clinical information obtained.

## Materials and methods

Study design

This study was structured as a retrospective data analysis. Ethical approval for this study was obtained from the Institutional Ethics Committee of Samsun University (SUKAEK-2023 8/7) and was registered to Clinicaltrials.gov with NCT06076096. Data collection for patients who underwent lower limb surgeries at Samsun University Education and Research Hospital during the period spanning from March 2022 to March 2023 was executed through the utilization of hospital automation systems (specifically, FONET v4.23.1.1, Turkey) and documentation extracted from perioperative follow-up forms. Patients classified within the American Society of Anesthesiology (ASA) categories I-I who underwent lower limb surgical procedures with the utilization of SIFIB either as the sole anesthesia method or as a constituent of the anesthesia plan were enrolled, and data were systematically gathered. Patients who received anesthesia management via general anesthesia, neuraxial anesthesia, or infiltration anesthesia were excluded. Written informed consent is an essential requirement for all procedures conducted as part of our clinical routine and obtained for all procedures. Due to the retrospective nature of this article, obtaining consent for publication was waived.

Data collection

Demographic data pertaining to patients, including their age, gender, weight, length, comorbidities, and ASA scores, were documented. Furthermore, the features of the surgical procedure, including its type and duration, as well as additional block or local anesthetic volume administered in milliliters, block onset time in minutes, total surgery duration in minutes, any supplementary analgesic or anesthetic requirements, and reported perioperative pain and its precise location, were all diligently recorded.

Block technique and routine analgesia protocol

SIFIB is administered approximately 30 minutes prior to the surgical procedure in a designated section within the operating room under standard ASA monitoring. We routinely use premedication prior to the block procedure as intravenous midazolam at a dosage of 0.01-0.02 mg/kg and fentanyl at 0.25 mcg/kg (or ketamine at 0.15 mg/kg).

For SIFIB, patients are placed supine, and all procedures are carried out in accordance with the rules of asepsis. SIFIB is performed under the guidance of a high-frequency linear transducer, and sciatic blocks are applied in the lateral position with a convex transducer (10-18 MHz and 3-5 MHz, respectively, Esoate MyLabTM30Gold, Genoa, Italy). In SIFIB, local anesthetic is injected into the compartment between the iliac fascia and iliacus muscle in the suprainguinal region, and the spread of local anesthetic is confirmed by US [7]. We routinely use 50 mL of the local anesthetic mixture in SIFIB and perform a frequent, systematic aspiration, effectively mitigating the possibility of unintended vascular injection. The local anesthetic mixture includes 0.5% bupivacaine (25 mL), 2% lidocaine (10 mL), and saline (15 mL). However, when applied together with a sciatic block, the bupivacaine concentration in the local anesthetic mixture used for SIFIB is reduced to 0.20%. For the sciatic block procedure, an additional 20 mL 0.25% of bupivacaine is used, and lidocaine is avoided as a precautionary measure to mitigate the risk of local anesthetic systemic toxicity.

In our clinical practice, propofol infusion at a dose of 1 mg/kg/hour is preferred to reduce the patient's awareness and maintain cooperation throughout the surgery and is adjusted according to whether the patient under conscious sedation reports pain. As a general rule, before increasing the dose of propofol, ketamine is administered in divided doses, with each dose being 10 mg, and these instances are meticulously recorded. Detailed records are also maintained for patients necessitating supplementary analgesics during the intraoperative phase, encompassing both the drug doses administered and the localization of pain.

Adhering to our standard clinical protocol, we consistently administer a standardized analgesic regimen. Each patient was managed with a multimodal analgesia approach involving the intravenous administration of both paracetamol (1 g) and tenoxicam (20 mg, intraoperatively. Paracetamol was administered at eight-hour intervals, while tenoxicam was administered every 12 hours.

Statistics

Data analysis was conducted using SPSS Statistics version 16 (SPSS Inc. Released 2007. SPSS for Windows, Version 16.0. Chicago, SPSS Inc.). Continuous quantitative data were expressed as numerical values along with the mean ± standard deviation. The chi-squared test followed by Yates' correction was used for categorical assessments, while the Mann-Whitney U test was employed for continuous data evaluations. A significance level of p<0.05 was established to determine statistical significance.

## Results

The data of a total of 16 patients were analyzed, including 10 patients (consisting of seven females and three males, with ages spanning from 43 to 78 years (mean age: 55.5±12.3 years) and body mass index (BMI) from 21.8 to 29.7 kg/m2 (mean BMI 27.0±2.81 kg/m2)) who underwent sole SIFIB and six patients (two males and four females aged between a minimum of 63 and a maximum of 81 years (mean age: 71.6±6.5 years) and with a BMI of 23.8 to 32.8 kg/m2 (mean BMI 29.6±3.4 kg/m2)) who underwent SIFIB combined with sciatic block. The surgical duration averaged 48.5±7.3 minutes. The onset time of the blocks was evaluated with a pinprick test by stimulating the anterolateral of the thigh and surgical incision area and was 29.1±5.1 minutes. In addition to patient demographic data, Table 1 presents parameters such as the type and duration of surgery, comorbidities, volume used for the block, onset time of the block, and the need for supplementary analgesia.

**Figure 1:**
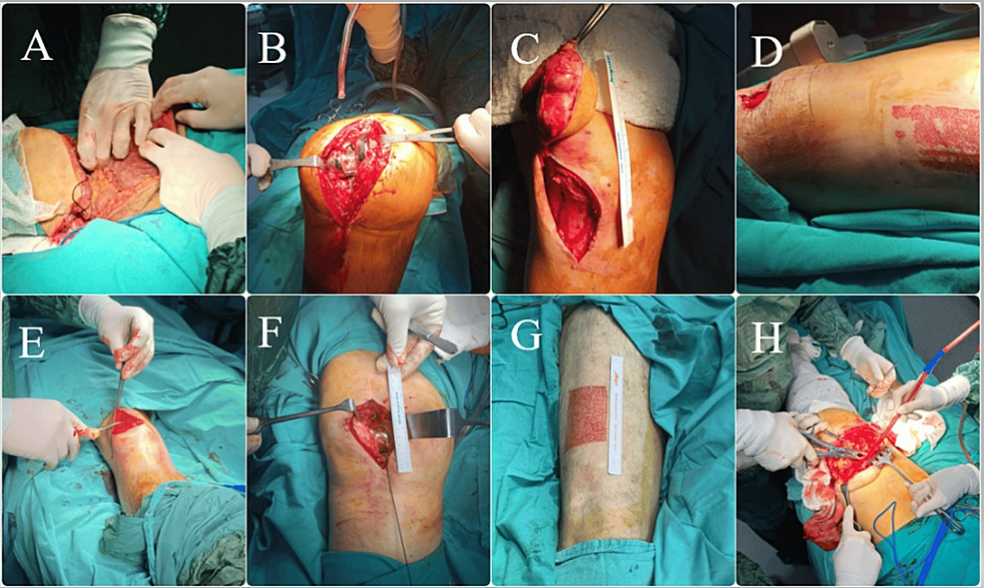
Surgical photographs of patients A: transfemoral knee amputation, B: revision of knee prosthesis, C: knee tumor excision, D: soft tissue excision from the knee and grafting, E: implant removal from the patella, F: patellar ligament repair, G: grafting, H: transfemoral knee amputation

The types of surgeries were above-knee amputations (transfemoral)(2), revision of knee prostheses, excision of knee tumors (2), soft tissue excision from the knee with grafting, implant removal from patella, patellar ligament repair, vein ligation and stripping (2), patellar fracture repair, grafting from the mid-thigh, distal femur fractures (3), and femoropopliteal bypass surgery (Table 1, Figure 1). In all patients, the blocks were administered unilaterally. In 10 cases, SIFIB was administered as a standalone procedure using a 50 mL local anesthetic mixture. In six patients, SIFIB was combined with a sciatic nerve block, using a subgluteal approach in transfemoral knee amputations (cases 11, 12) and a parasacral approach in distal femur fractures and femoropopliteal bypass surgery (cases 13-16). An additional 20 mL of a local anesthetic mixture (10 mL of 0.5% bupivacaine and 10 mL of saline) was used for the sciatic nerve block.

**Table 1:** Descriptives, block types, surgical procedure, and pain status of the patients HTN: hypertension, DM: diabetes mellitus, COPD: chronic obstructive pulmonary disease, CHD: coronary heart disease, CHF: congestive heart failure, SAH: subarachnoid hemorrhage, CRF: chronic renal failure, CVD: cerebrovascular disease, Fem-Pop: femoral popliteal bypass surgery, LA: local anesthetic, BMI: body mass index, NA: not available, ASA: American Society of Anesthesiology

Case number	Age/gender	BMI (kg/m^2^)	ASA comorbidities	Type and duration of surgical procedure	Additional block LA volume (mL)	Block onset time (minute)	Surgery duration (minute)	Additional analgesic/anesthetic requirement	Reported perioperative pain and its location
1	47/F	24.3	I / NA (patient’s request)	Knee tumor excision	Sole (50 mL)	33	31	10 mg ketamine	Mild pain at the inferomedial of the knee
2	53/F	29.2	III	Knee tumor excision	Sole (50 mL)	29	16	None	NA
3	65/F	25.3	III HTN, DM	Soft tissue excision from the knee and grafting	Sole (50 mL)	31	41	None	NA
4	43/F	21.8	III HTN, DM, COPD	Implant removal from patella	Sole (50 mL)	26	53	None	NA
5	78/F	29.0	III HTN, DM CHD, CHF (EF:%15)	Revision of knee prosthesis	Sole (50 mL)	31	41	10 mg ketamine	Mild/moderate pain at the posterior of the knee
6	73/F	29.7	III / HTN, DM, CHF, hyperthyroidism	Patellar ligament repair	Sole (50 mL)	32	41	None	NA
7	48/M	27.7	II heavy smoker HTN, COPD (patient’s request)	Vein ligation and stripping	Sole (50 mL)	30	71	None	NA
8	55/M	29.7	II / NA (patient’s request)	Vein ligation and stripping	Sole (50 mL)	33	42	None	NA
9	43/F	24.6	III / trauma, SAH, HTN	Patellar fracture	Sole (50 mL)	37	55	10 mg ketamine	Mild pain at the inferomedial of the knee
10	50/M	29.0	II / DM, heavy smoker	Grafting from mid-thigh	Sole (50 mL)	17	22	None	NA
11	75/M	31.8	III / HTN, DM, CRF, CVD	Transfemoral knee amputation	Sciatic block (+20 mL)	31	31	10 mg + 10 mg ketamine	Mild/moderate pain at the medial of thigh
12	75/M	23.8	III / HTN, DM, CRF	Transfemoral knee amputation	Sciatic block (+20 mL)	33	42	None	NA
13	68/F	26.7	III HTN, CHD	Femur fracture	Sciatic block (+20 mL)	21	36	None	NA
14	63/F	32.8	III DM, CVD	Femur fracture	Sciatic block (+20 mL)	26	31	10 mg ketamine	Mild/moderate pain at the medial of thigh
15	68/F	30.4	III HTN, CHF	Femur fracture	Sciatic block (+20 mL)	24	27	10 mg +10 mg ketamine	Mild/moderate pain at the medial of thigh
16	81/F	32.5	III heavy smoker, DM, COPD	Fem-Pop	Sciatic block (+20 mL)	32	56	None	NA

During these surgeries, one patient reported mild/moderate pain at the posterior of the knee (case 5), two patients reported mild pain on deep manipulation of the inferomedial of the knee (cases 1, 9), and three patients reported mild/moderate pain at the medial of thigh (cases 11, 14, 15). A very low dose of ketamine was given to these patients who reported pain during surgery (Table 1). No additional perioperative pain was documented. All surgical procedures were successfully completed without any complications. The postoperative monitoring throughout the day of surgery transpired without any noteworthy events for all patients, each of whom was discharged on the subsequent day.

When comparing patients who required sedoanalgesia during surgery (n:6) with those who did not (n:10), there were no statistically significant differences observed in terms of age, ASA classification, BMI, block onset time, and surgical duration (p>0.05). It is presented in Table 2.

**Table 2:** Patients' data necessitating sedoanalgesia during surgery, with those not requiring ASA classes were presented as the number of patients; all other data were presented as number, mean ± standard deviation, ASA: American Society of Anesthesiology, BMI: body mass index

	Need for additional sedoanalgesia (n:6)	No need for additional sedoanalgesia (n:10)	p-value
Age (years)	62.3±14.4	61.1±12.9	0.72
ASA classification I/II/III	1/0/5	0/3/7	-
BMI (kg/m^2^)	28.8±3.6	27.5±3.1	0.65
Block onset time (minutes)	30.3±4.7	28.4±5.4	0.79
Surgical duration (minutes)	36.0±10.4	42.0±15.8	0.37

## Discussion

Based on our clinical experience involving 16 patients who underwent lower extremity surgery for various indications, it is evident that the utilization of SIFIB or a combination of SIFIB and sciatic block can be an alternative option for surgical anesthesia.

US-guided SIFIB precisely targets the lumbar plexus components situated immediately medial to the spina iliaca anterior superior [8]. The act of blocking the obturator nerve in conjunction with the femoral nerve and lateral femoral cutaneous nerve can be perceived as an anterior and more superficial approach to the lumbar plexus. Nevertheless, controversy exists surrounding the mechanism of action concerning the dispersion of local anesthetic to the obturator nerve. The preference for SIFIB among clinicians can be attributed to its perceived superiority and ease of administration compared to conventional fascia iliaca blocks. Characterized by its relatively superficial nature, minimal risk of nerve or vascular injury during needle insertion, safe distance from critical circulatory structures, and the supine position's applicability, SIFIB can be employed as a secure primary anesthetic approach when administered with a relatively substantial volume of local anesthetic [4,5].

Indeed, the literature consistently highlights the use of SIFIB for postoperative analgesia in hip and knee surgeries as an integral component of a multimodal analgesic strategy [9-13]. Furthermore, studies report SIFIB as the main anesthetic method in selected patients. Azizoglu et al. [14] reported the successful attainment of surgical anesthesia through the utilization of SIFIB in conjunction with a sciatic nerve block in a high-risk patient undergoing surgery for a femur fracture, and Soulioti et al. [15] reported the use of SIFIB for surgical anesthesia in a patient undergoing emergency femoral thrombectomy.

Three patients reported intraoperative mild to moderate pain secondary to the surgical process extending beyond the planned area. Sciatic block or distal selective block applications could have been utilized to address this issue quickly. In case 1, the cause of the mild pain in the inferomedial patella was not due to insufficient obturator nerve blockage but failure to block the terminal branches originating from the sacral plexus of the superior and medial genicular nerves. Considering the complex innervation of the knee, the lumbar plexus block alone may not be enough for complete anesthesia. Similarly, the pain reported in case 5 was due to the area innervated by the sacral plexus. The interspace between the popliteal artery and capsule of the posterior knee block could have been utilized in this case; however, it was not predicted that the surgical procedure would extend to the ⅓ posterior aspect of the knee. It should be noted that in the selected patient group for whom transfemoral amputation will be performed, we are of the opinion that SIFIB+sciatic block may serve as an alternative to lumbar plexus block.

In high volume/low concentration SIFIB, the local anesthetic reaches the branches of the lumbar plexus more easily and leads to increased block effectiveness. SIFIB is generally performed using 40 mL of local anesthetic [16]; however, a recent cadaveric study reported the minimum effective volume (MEV90) for SIFIB as being 62.5 mL [17]. However, high fragility rates may be present in the selected/high-risk patient group who underwent high-volume SIFIB for surgical anesthesia. Additionally, it is known that the intercompartmental transfer of local anesthetic is high in elderly patients [18]. As such, these patients should be accepted as being at high risk for local anesthetic systemic toxicity, and therefore, additional care should be taken by clinicians. Koc et al. [19] conducted a study comparing two different concentrations of bupivacaine in SIFIB and found that both 0.20% and 0.25% concentrations exhibited similar levels of effectiveness. In six patients in whom a combination of SIFIB and sciatic nerve block was performed, even though the concentrations of bupivacaine and lidocaine were reduced, a total volume of 70 mL of local anesthetic was administered, and fortunately, no complications were observed. Furthermore, it should be noted that care should be taken to avoid puncture of the deep circumflex iliac artery, especially in high-risk patients who are receiving antiplatelet medication.

While anatomical variations may account for variability in pain perception among certain patients, individual patient characteristics also likely exerted influence. Factors such as the presence of chronic illnesses and an individual's recollection of prior pain experiences may be correlated with the perception and intensity of perioperative pain. Contemporary research endeavors are currently investigating the predictability of pain, with hemogram parameters being one of the areas under scrutiny [20,21]. It may be more logical for forthcoming studies to prioritize the investigation of pain predictability and consider block application as the primary analgesic approach in a homogenous patient population.

There are some limitations. The retrospective nature of the study heightens susceptibility to bias, especially considering the number of the study population. Feasibility was not assessed within homogeneous groups, considering the distinct innervation areas associated with each surgical procedure. When assessing surgical anesthesia methods for lower extremity surgery, it is essential to consider not only the surgical site but also the utilization of tourniquets. Anesthesia and analgesia management should be meticulously planned to accommodate these factors. Research studies should be conducted to investigate clinical, anatomical, and cutaneous mapping, specifically focusing on SIFIB and lumbar plexus block techniques.

## Conclusions

In patients scheduled for lower extremity surgery, SIFIB, as an alternative to lumbar plexus block, appears to be a feasible option that can be used alone or in combination with sciatic block. Of course, if these techniques or combinations are to be used as an alternative to general and neuraxial anesthesia, the cutaneous and osseous innervation areas and the possible blockage area of each technique should be taken into account on a per-procedural basis.
